# Supplementary material for: Resolving the full spectrum of human genome variation using Linked-Reads
Source: Genome Res. 2019 Apr;29(4):635–45. doi: 10.1101/gr.234443.118 (PMC6442396; doi:10.1101/gr.234443.118)
Supplement: Supplemental Material [file supp_gr.234443.118_Supplemental_longranger-2.2.zip › longranger-2.2.tar.bz2!/lr2.2-public/lib/stan/cmdstan/stan/doc/api/html/multi__normal__cholesky_8hpp_source.html]

Stan: src/stan/prob/distributions/multivariate/continuous/multi\_normal\_cholesky.hpp Source File


|  |  |
| --- | --- |
|  | Stan  2.5.0  probability, sampling & optimization |


- Main Page
- Related Pages
- Namespaces
- Classes
- Files

- File List
- File Members

All Classes Namespaces Files Functions Variables Typedefs Enumerations Enumerator Friends Macros Pages

- src
- stan
- prob
- distributions
- multivariate
- continuous

multi\_normal\_cholesky.hpp

Go to the documentation of this file.

1 #ifndef STAN\_\_PROB\_\_DISTRIBUTIONS\_\_MULTIVARIATE\_\_CONTINUOUS\_\_MULTI\_NORMAL\_CHOLESKY\_HPP

2 #define STAN\_\_PROB\_\_DISTRIBUTIONS\_\_MULTIVARIATE\_\_CONTINUOUS\_\_MULTI\_NORMAL\_CHOLESKY\_HPP

3

4 #include <boost/random/normal\_distribution.hpp>

5 #include <boost/random/variate\_generator.hpp>

6

7 #include <boost/random/normal\_distribution.hpp>

8 #include <boost/random/variate\_generator.hpp>

9

10 #include <stan/agrad/rev.hpp>

11 #include <stan/agrad/rev/matrix.hpp>

12 #include <stan/math/error\_handling.hpp>

13 #include <stan/math/matrix/columns\_dot\_product.hpp>

14 #include <stan/math/matrix/columns\_dot\_self.hpp>

15 #include <stan/math/matrix/dot\_product.hpp>

16 #include <stan/math/matrix/dot\_self.hpp>

17 #include <stan/math/matrix\_error\_handling.hpp>

18 #include <stan/math/matrix/log.hpp>

19 #include <stan/math/matrix/log\_determinant.hpp>

20 #include <stan/math/matrix/mdivide\_left\_spd.hpp>

21 #include <stan/math/matrix/mdivide\_left\_tri\_low.hpp>

22 #include <stan/math/matrix/multiply.hpp>

23 #include <stan/math/matrix/subtract.hpp>

24 #include <stan/math/matrix/sum.hpp>

25 #include <stan/meta/traits.hpp>

26 #include <stan/prob/constants.hpp>

27 #include <stan/prob/traits.hpp>

28

29 namespace stan {

30

31  namespace prob {

32

50  template <bool propto,

51  typename T\_y, typename T\_loc, typename T\_covar>

52  typename boost::math::tools::promote\_args<typename scalar\_type<T\_y>::type, typename scalar\_type<T\_loc>::type, T\_covar>::type

53  multi\_normal\_cholesky\_log(const T\_y& y,

54  const T\_loc& mu,

55  const Eigen::Matrix<T\_covar,Eigen::Dynamic,Eigen::Dynamic>& L) {

56  static const char\* function = "stan::prob::multi\_normal\_cholesky\_log(%1%)";

57  typedef typename boost::math::tools::promote\_args<typename scalar\_type<T\_y>::type, typename scalar\_type<T\_loc>::type, T\_covar>::type lp\_type;

58  lp\_type lp(0.0);

59

60  using stan::math::mdivide\_left\_tri\_low;

61  using stan::math::dot\_self;

62  using stan::math::multiply;

63  using stan::math::subtract;

64  using stan::math::sum;

65

66  using stan::math::check\_size\_match;

67  using stan::math::check\_finite;

68  using stan::math::check\_not\_nan;

69

70  VectorViewMvt<const T\_y> y\_vec(y);

71  VectorViewMvt<const T\_loc> mu\_vec(mu);

72  //size of std::vector of Eigen vectors

73  size\_t size\_vec = max\_size\_mvt(y, mu);

74

75  //Check if every vector of the array has the same size

76  int size\_y = y\_vec[0].size();

77  int size\_mu = mu\_vec[0].size();

78  if (size\_vec > 1) {

79  int size\_y\_old = size\_y;

80  int size\_y\_new;

81  for (size\_t i = 1, size\_ = length\_mvt(y); i < size\_; i++) {

82  int size\_y\_new = y\_vec[i].size();

83  check\_size\_match(function,

84  size\_y\_new, "Size of one of the vectors of the random variable",

85  size\_y\_old, "Size of another vector of the random variable",

86  &lp);

87  size\_y\_old = size\_y\_new;

88  }

89  int size\_mu\_old = size\_mu;

90  int size\_mu\_new;

91  for (size\_t i = 1, size\_ = length\_mvt(mu); i < size\_; i++) {

92  int size\_mu\_new = mu\_vec[i].size();

93  check\_size\_match(function,

94  size\_mu\_new, "Size of one of the vectors of the location variable",

95  size\_mu\_old, "Size of another vector of the location variable",

96  &lp);

97  size\_mu\_old = size\_mu\_new;

98  }

99  (void) size\_y\_old;

100  (void) size\_y\_new;

101  (void) size\_mu\_old;

102  (void) size\_mu\_new;

103  }

104

105  check\_size\_match(function,

106  size\_y, "Size of random variable",

107  size\_mu, "size of location parameter",

108  &lp);

109  check\_size\_match(function,

110  size\_y, "Size of random variable",

111  L.rows(), "rows of covariance parameter",

112  &lp);

113  check\_size\_match(function,

114  size\_y, "Size of random variable",

115  L.cols(), "columns of covariance parameter",

116  &lp);

117

118  for (size\_t i = 0; i < size\_vec; i++) {

119  check\_finite(function, mu\_vec[i], "Location parameter", &lp);

120  check\_not\_nan(function, y\_vec[i], "Random variable", &lp);

121  }

122

123  if (size\_y == 0)

124  return lp;

125

126  if (include\_summand<propto>::value)

127  lp += NEG\_LOG\_SQRT\_TWO\_PI \* size\_y \* size\_vec;

128

129  if (include\_summand<propto,T\_covar>::value)

130  lp -= L.diagonal().array().log().sum() \* size\_vec;

131

132  if (include\_summand<propto,T\_y,T\_loc,T\_covar>::value) {

133  lp\_type sum\_lp\_vec(0.0);

134  for (size\_t i = 0; i < size\_vec; i++) {

135  Eigen::Matrix<typename

136  boost::math::tools::promote\_args<typename scalar\_type<T\_y>::type, typename scalar\_type<T\_loc>::type>::type,

137  Eigen::Dynamic, 1> y\_minus\_mu(size\_y);

138  for (int j = 0; j < size\_y; j++)

139  y\_minus\_mu(j) = y\_vec[i](j)-mu\_vec[i](j);

140  Eigen::Matrix<typename

141  boost::math::tools::promote\_args<T\_covar,typename scalar\_type<T\_loc>::type,typename scalar\_type<T\_y>::type>::type,

142  Eigen::Dynamic, 1>

143  half(mdivide\_left\_tri\_low(L,y\_minus\_mu));

144  // FIXME: this code does not compile. revert after fixing subtract()

145  // Eigen::Matrix<typename

146  // boost::math::tools::promote\_args<T\_covar,typename scalar\_type<T\_loc>::type,typename scalar\_type<T\_y>::type>::type>::type,

147  // Eigen::Dynamic, 1>

148  // half(mdivide\_left\_tri\_low(L,subtract(y,mu)));

149  sum\_lp\_vec += dot\_self(half);

150  }

151  lp -= 0.5\*sum\_lp\_vec;

152  }

153  return lp;

154  }

155

156  template <typename T\_y, typename T\_loc, typename T\_covar>

157  inline

158  typename boost::math::tools::promote\_args<typename scalar\_type<T\_y>::type, typename scalar\_type<T\_loc>::type, T\_covar>::type

159  multi\_normal\_cholesky\_log(const T\_y& y,

160  const T\_loc& mu,

161  const Eigen::Matrix<T\_covar,Eigen::Dynamic,Eigen::Dynamic>& L) {

162  return multi\_normal\_cholesky\_log<false>(y,mu,L);

163  }

164

165  template <class RNG>

166  inline Eigen::VectorXd

167  multi\_normal\_cholesky\_rng(const Eigen::Matrix<double,Eigen::Dynamic,1>& mu,

168  const Eigen::Matrix<double,Eigen::Dynamic,Eigen::Dynamic>& S,

169  RNG& rng) {

170  using boost::variate\_generator;

171  using boost::normal\_distribution;

172

173  static const char\* function = "stan::prob::multi\_normal\_cholesky\_rng(%1%)";

174

175  using stan::math::check\_finite;

176

177  check\_finite(function, mu, "Location parameter", (double\*)0);

178

179  variate\_generator<RNG&, normal\_distribution<> >

180  std\_normal\_rng(rng, normal\_distribution<>(0,1));

181

182  Eigen::VectorXd z(S.cols());

183  for(int i = 0; i < S.cols(); i++)

184  z(i) = std\_normal\_rng();

185

186  return mu + S \* z;

187  }

188  }

189 }

190

191 #endif

columns\_dot\_self.hpp

matrix.hpp

mdivide\_left\_tri\_low.hpp

stan::math::mdivide\_left\_tri\_low

Eigen::Matrix< typename boost::math::tools::promote\_args< T1, T2 >::type, R1, C2 > mdivide\_left\_tri\_low(const Eigen::Matrix< T1, R1, C1 > &A, const Eigen::Matrix< T2, R2, C2 > &b)

**Definition:** mdivide\_left\_tri\_low.hpp:16

stan::agrad::dot\_self

fvar< T > dot\_self(const Eigen::Matrix< fvar< T >, R, C > &v)

**Definition:** dot\_self.hpp:16

stan::math::dot\_self

double dot\_self(const std::vector< double > &x)

**Definition:** dot\_self.hpp:11

stan::prob::multi\_normal\_cholesky\_rng

Eigen::VectorXd multi\_normal\_cholesky\_rng(const Eigen::Matrix< double, Eigen::Dynamic, 1 > &mu, const Eigen::Matrix< double, Eigen::Dynamic, Eigen::Dynamic > &S, RNG &rng)

**Definition:** multi\_normal\_cholesky.hpp:167

stan::max\_size\_mvt

size\_t max\_size\_mvt(const T1 &x1, const T2 &x2)

**Definition:** traits.hpp:575

traits.hpp

stan::prob::multi\_normal\_cholesky\_log

boost::math::tools::promote\_args< typename scalar\_type< T\_y >::type, typename scalar\_type< T\_loc >::type, T\_covar >::type multi\_normal\_cholesky\_log(const T\_y &y, const T\_loc &mu, const Eigen::Matrix< T\_covar, Eigen::Dynamic, Eigen::Dynamic > &L)

The log of the multivariate normal density for the given y, mu, and a Cholesky factor L of the varian...

**Definition:** multi\_normal\_cholesky.hpp:53

columns\_dot\_product.hpp

stan::math::check\_size\_match

bool check\_size\_match(const char \*function, T\_size1 i, const char \*name\_i, T\_size2 j, const char \*name\_j, T\_result \*result)

**Definition:** check\_size\_match.hpp:13

error\_handling.hpp

mdivide\_left\_spd.hpp

stan::math::check\_finite

bool check\_finite(const char \*function, const T\_y &y, const char \*name, T\_result \*result)

Checks if the variable y is finite.

**Definition:** check\_finite.hpp:52

log.hpp

stan::scalar\_type::type

scalar\_type\_helper< is\_vector< T >::value, T >::type type

**Definition:** traits.hpp:139

matrix\_error\_handling.hpp

constants.hpp

traits.hpp

dot\_self.hpp

stan::prob::include\_summand

Template metaprogram to calculate whether a summand needs to be included in a proportional (log) prob...

**Definition:** traits.hpp:35

stan::math::subtract

Eigen::Matrix< typename boost::math::tools::promote\_args< T1, T2 >::type, R, C > subtract(const Eigen::Matrix< T1, R, C > &m1, const Eigen::Matrix< T2, R, C > &m2)

Return the result of subtracting the second specified matrix from the first specified matrix...

**Definition:** subtract.hpp:27

rev.hpp

log\_determinant.hpp

stan::length\_mvt

size\_t length\_mvt(const T &)

**Definition:** traits.hpp:561

dot\_product.hpp

stan::math::sum

double sum(std::vector< double > &x)

**Definition:** sum.hpp:10

stan::math::check\_not\_nan

bool check\_not\_nan(const char \*function, const T\_y &y, const char \*name, T\_result \*result)

Checks if the variable y is nan.

**Definition:** check\_not\_nan.hpp:56

size\_

size\_t size\_

**Definition:** dot\_self.hpp:18

stan::VectorViewMvt

**Definition:** traits.hpp:514

stan::math::multiply

boost::enable\_if\_c< boost::is\_arithmetic< T >::value, Eigen::Matrix< double, R, C > >::type multiply(const Eigen::Matrix< double, R, C > &m, T c)

Return specified matrix multiplied by specified scalar.

**Definition:** multiply.hpp:25

subtract.hpp

stan::agrad::mdivide\_left\_tri\_low

Eigen::Matrix< fvar< T >, R1, C1 > mdivide\_left\_tri\_low(const Eigen::Matrix< fvar< T >, R1, C1 > &A, const Eigen::Matrix< fvar< T >, R2, C2 > &b)

**Definition:** mdivide\_left\_tri\_low.hpp:22

sum.hpp

multiply.hpp


---

[ Stan Home Page ]

*© 2011–2014,
Stan Development Team.*
